# Modifications decrease hepatic steatosis in Taiwanese with metabolic‐associated fatty liver disease

**DOI:** 10.1002/kjm2.12580

**Published:** 2022-08-22

**Authors:** Tso‐Tsai Liu, He Qiu, Shi‐Yu Liu, Chieh Chien, Jen‐Hung Wang, Ming‐Wun Wong, Chih‐Hsun Yi, Lin Lin, Wei‐Yi Lei, Shu‐Wei Liang, Jui‐Sheng Hung, Jee‐Fu Huang, Chien‐Lin Chen, Ma Ai Thanda Han

**Affiliations:** ^1^ Department of Medicine, Hualien Tzu Chi Hospital Buddhist Tzu Chi Medical Foundation and Tzu Chi University Hualien Taiwan; ^2^ Division of Gastroenterology and Hepatology, Department of Medicine Rutgers New Jersey Medical School Newark New Jersey USA; ^3^ Department of Nutrition, Hualien Tzu Chi Hospital Buddhist Tzu Chi Medical Foundation and Tzu Chi University Hualien Taiwan; ^4^ Department of Rehabilitation Medicine, Hualien Tzu Chi Hospital Buddhist Tzu Chi Medical Foundation and Tzu Chi University Hualien Taiwan; ^5^ Department of Medical Research, Hualien Tzu Chi Hospital Buddhist Tzu Chi Medical Foundation Hualien Taiwan; ^6^ School of Post‐Baccalaureate Chinese Medicine Tzu Chi University Hualien Taiwan; ^7^ Hepatobiliary Division, Department of Internal Medicine Kaohsiung Medical University Hospital Kaohsiung Taiwan; ^8^ Institute of Medical Sciences Tzu Chi University Hualien Taiwan; ^9^ Division of Gastroenterology and Hepatology, Department of Medicine Banner University Medical Center, University of Arizona Phoenix Arizona USA

**Keywords:** fatty liver, lifestyle intervention, nonalcoholic fatty liver disease, nonalcoholic steatohepatitis, weight loss

## Abstract

Metabolic‐associated fatty liver disease (MAFLD) is a growing global problem associated with increasing obesity prevalence. Lifestyle modifications are currently recommended, including weight reduction, exercise, and diet control. This study evaluated the short‐term effect of lifestyle modifications on transient elastography (TE) values in an obese population with MAFLD. Thirty‐two MAFLD patients were recruited for this prospective study and all subjects participated in a 3‐month program of lifestyle modification. Sequential demographic parameters and biochemical tests were compared before and after program completion. Liver fat and fibrosis changes were measured using TE with controlled attenuated parameter (CAP) and liver stiffness measurements (LSM). The mean age was 38.7 years old (10 males). The body weight (88.09 kg vs. 80.35 kg), body mass index (32.24 kg/m^2^ vs. 29.4 kg/m^2^), waist (103.19 cm vs. 95.75 cm), and hip circumference (111.67 cm vs. 104.75 cm), and blood pressure (128/78 mmHg vs. 119/71 mmHg) significantly improved before and after the intervention, respectively. Aspartate aminotransaminase (24.06 U/L vs. 18.91 U/L), alanine aminotransaminase (33 U/L vs. 23.72 U/L), creatinine (0.75 mg/dl vs. 0.70 mg/dl), cholesterol (176.41 mg/dl vs. 166.22 m/dl), gamma‐glutamyl transferase (26.59 IU/L vs. 19.81 IU/L), and low‐density lipoprotein cholesterol (115.63 mg/dl vs. 103.19 mg/dl) also improved after the 3‐month intervention. The average CAP significantly decreased after intervention (297.5 dB/m vs. 255.0 dB/m), however, no significant difference in LSM was observed (5.24 kPa vs. 4.82 kPa). The current study suggests that short‐term lifestyle modification can effectively improve hepatic steatosis, and TE may serve as a monitoring tool for therapeutic intervention.

## 1 INTRODUCTION

Metabolic‐associated fatty liver disease (MAFLD), a new acronym adopted from international expert consensus, is the most prevalent liver disease worldwide. It is defined based on the presence of excessive fat accumulation in the liver in addition to obesity, presence of type 2 diabetes mellitus (T2DM), or evidence of metabolic syndrome, regardless of alcohol consumption or concomitant underlying chronic liver disease.^1^ Besides cardiovascular risk, these patients are also at risk for fibrogenesis and cirrhosis with disease progression, which in turn, results in liver‐associated complications (e.g., portal hypertension, varices, and hepatocellular carcinoma).^2^

No approved drugs for MAFLD currently exist. In addition, the disease course remains heterogeneous and individualized. The key to managing MAFLD patients consists of weight loss and treating the underlying metabolic comorbidities including T2DM, obesity, hypertension, and hyperlipidemia. Previous studies have shown that lifestyle modification significantly reduced weight, reduced liver fat and inflammation, and improved aminotransferase levels in patients with nonalcoholic fatty liver disease (NAFLD).^3^, ^4^, ^5^ Therefore, the efficacy of lifestyle modification in MAFLD patients warrants further validation in a clinical setting.

Liver biopsy remains the gold standard for assessing liver steatosis and fibrosis; however, problems (e.g., invasiveness, cost, and sampling error) limit its practicality in diagnosing patients with fatty liver disease.^6^, ^7^, ^8^ Several noninvasive biomarkers that utilize clinical, biochemical, and metabolic parameters to delineate simple steatosis from steatohepatitis or advanced fibrosis have been proposed. In addition, noninvasive imaging tests, for example, transient elastography (TE) and magnetic resonance elastography, have also been studied and shown promising sensitivity and specificity for detecting advanced fibrosis in patients with NAFLD.^9^, ^10^

Nonetheless, limited studies evaluating the feasibility of TE in monitoring the degree of steatosis and fibrosis in patients with MAFLD undergoing lifestyle modifications were noted. Therefore, the effect of exercise and diet modification on controlled attenuated parameter (CAP) values in a group of obese patients with MAFLD was explored in this study.

## 2 METHODS

### 2.1 Subjects

**FIGURE 1:**
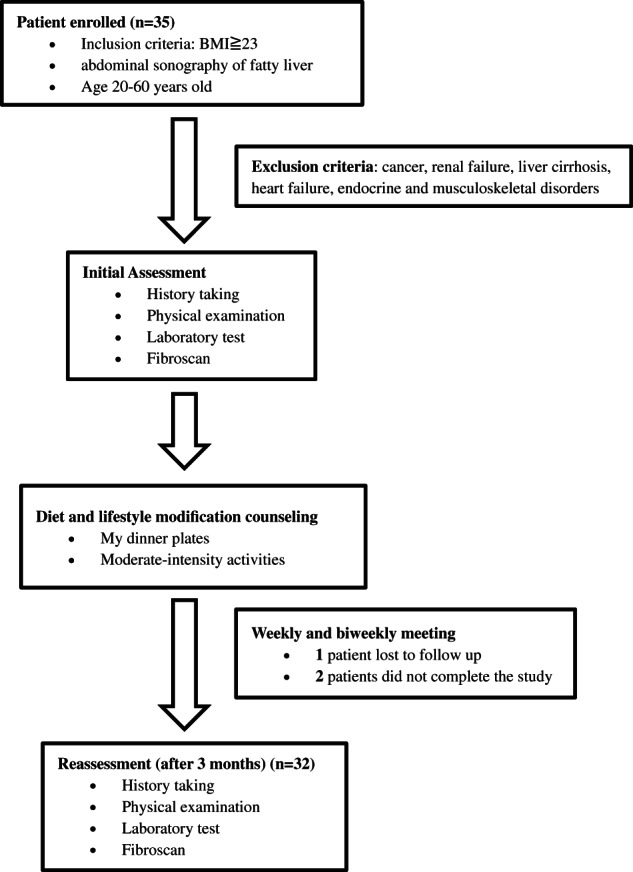
Flow chart of patient selection and study design

This study (conducted from March 2020 to October 2020) recruited 35 MAFLD patients aged between 18 and 60 years old who had a body mass index (BMI) of ≧23 kg/m^2^ with evidence of fatty liver disease by abdominal ultrasound, regardless of the presence of metabolic syndrome, from the outpatient clinic of the Department of Gastrointestinal Hepatobiliary at Hualien Tzu Chi Hospital, Buddhist Tzu Chi Medical Foundation, Hualien, Taiwan (Figure 1). All study participants were nonsmokers. Patients who had a history of cancer, renal failure, clinical evidence of liver cirrhosis, heart failure, and musculoskeletal disorders that could affect physical activity, were excluded from the study. The participants were followed for 3 months. This study was approved by the Research Ethical Committee of Hualien Tzu Chi Hospital, Buddhist Tzu Chi Medical Foundation, Hualien, Taiwan. All patients provided informed consent before study entry.

### 2.2 Study design

#### 2.2.1 Assessment

Each patient was measured for baseline height, weight, waist, and hip circumferences, body and visceral fats, skeletal muscle composition (HBF‐702 T Omron Healthcare Co. Ltd., Kyoto, Japan), blood pressure, heart rate, and respiration rate. Biochemical parameters included alanine aminotransaminase (ALT), aspartate aminotransaminase (AST), lipid profile, renal function, fasting and postprandial glucose, hemoglobin A1c (HbA1c), and homeostasis model assessment for insulin resistance before the beginning and after 12 weeks of the program. At the end of the study, the goal of the body weight reduction is 0.5–1 kg per week or 5%–10% weight loss within the studied period. The secondary goal is the improvement of hepatis steatosis and steatosis grade assessed by TE.

LSM and CAP were performed using TE (FibroScan 530 Compact, Echosens, Paris, France) scanning by a trained hepatologist at the initial assessment and the end of the exercise program. The current study used the following cutoffs for steatosis (S0, <215; S1, 215–252; S2, 253–296; and S3, >296) and fibrosis stage (F0, <7; F1–2, 7–8.6; F3, 8.7–10.2; and F4, >10.2) following the manufacturer's instructions and previous studies.^11^, ^12^

### 2.3 Intervention program

#### 2.3.1 Lifestyle intervention

The intervention focuses on changing both eating and exercise habits to reduce weight by 5%–10% within the study period. All participants were instructed to adhere to the advice of their physician concerning dietary control and physical activity. The exercise programs were conducted in the outpatient physiotherapy clinic, Hualien Tzu Chi Hospital, and were monitored by trained physiotherapists. Each participant met with the dietician and physiotherapist weekly during the first month and on a biweekly basis in subsequent months.

#### 2.3.2 Diet

A qualified dietician calculated the total daily calorie requirement for each participant according to their ideal body weight, basal metabolic rate, stress, and activity index. The daily calorie requirement was then sequentially reduced by 500–1000 kcal weekly until reaching their basal metabolic rate per day. They were instructed to consume a healthy diet and avoid foods with added sugars, processed meats, and refined grains (e.g., white bread, white pasta, and pizza). The food control was based on the concept of *my dinner plate*. Six types of foods that should be consumed daily include whole grains, beans, fish, eggs, vegetables, fruits, dairy products, and nut seeds. These foods were allocated to the daily meal following the daily calorie intake.

#### 2.3.3 Physical activity

The program emphasized moderate‐intensity activities (e.g., walking at least 10,000 steps per day). Other aerobic activities (e.g., jogging, bicycling, swimming, and dancing) were also recommended. Participants were instructed to have at least 150 min of these activities per week, with at least 30 min per day and five times per week.

### 2.4 Statistical analysis

Statistical analyses were conducted using SPSS version 22.0 software (SPSS Inc., Chicago, IL, USA). Comparison between pre and postintervention on relevant baseline variables and demographic characteristics were analyzed using paired *t* test and Pearson's chi‐square test for continuous and categorical variables, respectively. The steatosis grade improvement was analyzed with Stuart‐Maxwell test. The factors associated with improvement in hepatic steatosis were analyzed using univariate and multivariate logistic regression, adjusted for demographics, anthropometrics, and co‐morbidities. The results were considered statistically significant for *p* < .05 with a 95% confidence interval. For sample size estimation, an initial power analysis was applied (*F* test with a statistical power of 0.80, α error = .05, and effect size = 0.6). At least 25 number of pairs were required for this study. All data are presented as *n* or the mean ± SD.

## 3 RESULTS

### 3.1 Patient characteristics

**FIGURE 2:**
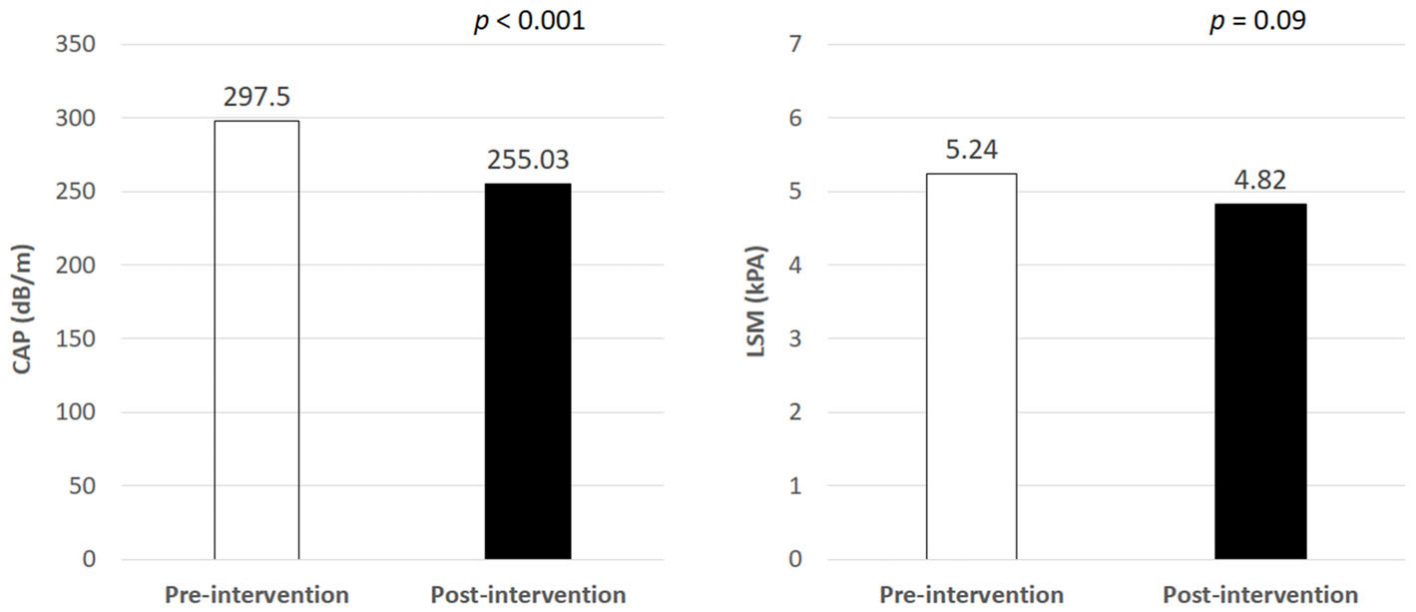
Controlled attenuation parameter (CAP) and liver stiffness (LSM) values before and after intervention

Thirty‐five participants were enrolled in the study with 32 (91%) patients completing the study. Three participants either did not complete the study or were lost to follow‐up during the 3 months. The baseline characteristics of the participants are shown in Table 1. This study had more females than males (69% vs. 31%). Six (18.8%) patients had underlying hypertension, 7 (21.9%) patients had hyperlipidemia, and 5 (15.6%) patients had chronic hepatitis B infection. All the patients with hyperlipidemia were taking the lipid‐lowering drugs during the entire duration of the study. There was no patient with diabetes mellitus, hepatitis C infection or excessive alcohol consumption (defined as >3 drinks per day in men and >2 drinks per day in women). The mean age of the participants was 38.7 years old. Before the intervention, the mean BMI, CAP, and LSM of the patients were 32.24 kg/m^2^, 297.5 dB/m, and 5.24 kPa, respectively (Figure 2). Majority of the patients (53.1%) has steatosis score of S3 prior to intervention, followed by S1 (28.1%) and S2 (18.8%) (Table 1).

**TABLE 1:** Baseline characteristics and anthropometrics

	Total	Male	Female
Sex, *n* (%)	32	10 (31.3)	22 (68.7)
Comorbidities			
Hypertension, *n* (%)	6	3 (50)	3 (50)
Hyperlipidemia, *n* (%)	7	2 (28.6)	5 (71.4)
Chronic hepatitis B infection (%)	5	1 (20)	4 (80)
Age	38.72 ± 8.89	37.7 ± 10.35	39.18 ± 8.36
Waist (cm)	103.19 ± 13.12	112.75 ± 13.21	98.84 ± 10.77
Hip (cm)	111.67 ± 10.72	118.3 ± 10.88	108.66 ± 9.41
Weight (kg)	88.09 ± 20.75	111.34 ± 16.75	77.52 ± 11.86
WHR	0.92 ± 0.06	0.95 ± 0.05	0.91 ± 0.05
Body Fat (%)	37.07 ± 4.18	33.93 ± 4.98	38.49 ± 2.9
BMI (kg/m^2^)	32.24 ± 4.98	35.5 ± 4.87	30.76 ± 4.38
SBP (mmHg)	127.66 ± 15.57	139.1 ± 14.61	122.45 ± 13.25
DBP (mmHg)	78.03 ± 13.58	84.3 ± 17.81	75.18 ± 10.44
Heart rate (BPM)	84.5 ± 11.45	84.6 ± 13.62	84.45 ± 10.68
Hepatic steatosis score (S), *n* (%)			
S1 (215–252)	9 (28.1)	2 (20)	7 (31.8)
S2 (253–296)	6 (18.8)	0 (0)	6 (27.3)
S3 (>296)	17 (53.1)	8 (80)	9 (40.9)

*Note*: Data are presented as *n* or mean ± SD.

Abbreviations: BMI, body mass index; DBP, diastolic blood pressure; SBP, systolic blood pressure; WHR, waist‐hip ratio.

### 3.2 Reduction of weight and comorbidities

**FIGURE 3:**
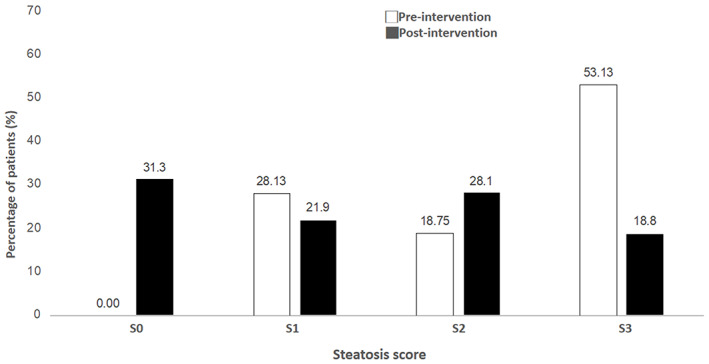
Distribution of steatosis score before and after intervention

The mean weight change over 3 months was −7.44 kg. (Table 2). The average body fat and BMI was reduced by 2.8% and 2.84 kg/m^2^, respectively. The mean waist and hip circumference changes were −7.44 (*p* < .001) and −6.92 (*p* < .001), respectively. Resting systolic (127.66 ± 15.57 vs. 119.09 ± 12.02, *p* < .001) and diastolic (78.03 ± 13.58 vs. 71.09 ± 8.29, *p* = .006) blood pressure as well as heart rate (84.5 ± 11.45 vs. 76.03 ± 10.91, *p* < .001) after intervention were also significantly reduced compared with before intervention. A significant reduction in CAP was observed after intervention (297.5 dB/m vs. 255 dB/m, *p* < .001). No significant differences in liver stiffness scores were noted when the scores were compared between before and after intervention (5.24 kPa vs. 4.82 kPa, *p* = .09; Figure 2). The distribution of steatosis severity is shown in Figure 3. The proportion of severe steatosis (S3) significant reduced after intervention (53.1% vs. 18.8%). Approximately one‐third (31.3%) patients achieved reversal of hepatic steatosis at the end of the study. In addition, 21 (65.6%) patients showed at least one grade improvement of hepatic steatosis (*p* = .001, Table S1). When analyzing the factor associated with improvement of hepatic steatosis, gender was the only significant factor. Male patients were associated with significantly lower odds ratio (OR = 0.07 [0.01, 0.92], *p* = .043; Table 3).

**TABLE 2:** Comparison of parameters before and after intervention

	Preintervention	Postintervention	Difference	*p* value
Waist (cm)	103.19 ± 13.12	95.75 ± 11.96	−7.44 ± 3.72	<.001
Hip (cm)	111.67 ± 10.72	104.75 ± 10.55	−6.92 ± 3.27	<.001
Weight (kg)	88.09 ± 20.75	80.35 ± 19.51	−7.74 ± 3.97	<.001
WHR	0.92 ± 0.06	0.91 ± 0.05	−0.01 ± 0.02	.026
Body fat (%)	37.07 ± 4.18	34.27 ± 4.44	−2.8 ± 1.44	<.001
BMI (kg/m^2^)	32.24 ± 4.98	29.4 ± 4.72	−2.84 ± 1.35	<.001
SBP (mmHg)	127.66 ± 15.57	119.09 ± 12.02	−8.56 ± 10.61	<.001
DBP (mmHg)	78.03 ± 13.58	71.09 ± 8.29	−6.94 ± 13.23	.006
Heart rate (BPM)	84.5 ± 11.45	76.03 ± 10.91	−8.47 ± 12.06	<.001

*Note*: Data are presented as *n* or mean ± SD.

Abbreviations: BMI, body mass index; DBP, diastolic blood pressure; SBP, systolic blood pressure; WHR, waist‐hip ratio.

**TABLE 3:** Factors associated with improvement of the severity of hepatic steatosis

	Unadjusted		Adjusted	
	OR (95% CI)	*p* value	OR (95% CI)	*p* value
Age	1.05 (0.96, 1.14)	.297	1.14(0.95, 1.37)	.145
Gender (male)	0.10 (0.02, 0.54)	.008	0.07 (0.01, 0.92)	.043
BMI	0.87 (0.74, 1.02)	.092	0.99 (0.74, 1.34)	.963
Preintervention CAP	1.00 (0.98, 1.01)	.717	1.01 (0.99, 1.04)	.365
HBV	0.75 (0.11, 5.32)	.774	0.41 (0.03, 6.76)	.536
HTN	0.29 (0.05, 1.65)	.163	0.08 (0.001, 4.04)	.203
HLD	3.12 (0.32, 30.79)	.329	2.70 (0.11, 64.45)	.540

Abbreviations: BMI, body mass index; CAP, controlled attenuated parameter; HBV, hepatitis B infection; HLD, hyperlipidemia; HTN, hypertension; OR, odds ratio.

At the end of the study, one patient stopped the hypertensive drug completely, and the other five patients achieved dose reduction. Two patients stopped the lipid‐lowering medication completely, and the other five patients continued the same medication at same dosage.

### 3.3 Improvement of biochemical parameters

No significant differences in HbA1c, glucose, blood urea nitrogen, triglyceride, high‐density lipoprotein levels, or homeostatic model assessment were noted for insulin resistance (HOMA‐IR; Table 4). The mean reduction in ALT and AST was −9.28 U/L (*p* = .012) and − 5.16 U/L (*p* = .002), respectively. Creatinine (0.75 mg/dl vs. 0.70 mg/dl, *p* = .002), total cholesterol (176.41 mg/dl vs. 166.22 m/dl, *p* = 0.023), low‐density lipoprotein (115.63 mg/dl vs. 103.19 mg/dl, *p* = .002), and gamma‐glutamyl transferase (26.59 IU/L vs. 19.81 IU/L, *p* = .001) levels were significantly reduced after intervention.

**TABLE 4:** Comparison in biochemical testing before and after intervention

	Preintervention	Postintervention	Difference	*p* value
HbA1c (%)	5.44 ± 0.54	5.47 ± 0.36	0.03 ± 0.33	.67
Fasting Glucose (mg/dl)	89.44 ± 8.06	92.22 ± 7.56	2.78 ± 6.59	.023
AST (U/L)	24.06 ± 8.85	18.91 ± 6.4	−5.16 ± 8.76	.002
ALT (U/L)	33 ± 20.76	23.72 ± 14.72	−9.28 ± 19.79	.012
GGT (IU/L)	26.59 ± 18.23	19.81 ± 14.83	−6.78 ± 10.05	.001
BUN (mg/dl)	11.09 ± 2.23	11.31 ± 2.29	0.22 ± 2.55	.631
Creatinine (mg/dl)	0.75 ± 0.17	0.70 ± 0.14	−0.05 ± 0.08	.002
Chol (mg/dl)	176.41 ± 31.32	166.22 ± 32.13	−10.19 ± 24.14	.023
TG (mg/dl)	122.59 ± 49.11	113.28 ± 61.32	−9.31 ± 46.8	.269
HDL (mg/dl)	42.88 ± 8.29	43.94 ± 8.86	1.06 ± 4.99	.238
LDL (mg/dl)	115.63 ± 28.03	103.19 ± 29.83	−12.44 ± 20.59	.002
HOMA‐IR (mg/dl)	2.65 ± 1.61	2.45 ± 1.85	−0.2 ± 1.94	.564

*Note*: Data are presented as *n* or mean ± SD.

Abbreviations: ALT, alanine aminotransferase; AST, aspartate aminotransferase; BUN, blood urea nitrogen; Chol, total cholesterol; GGT, gamma‐glutamyl transferase; HbA1c, hemoglobin A1c; HDL, high‐density liproprotein; HOMA‐IR, homeostatic model assessment for insulin resistance; LDL, low‐density lipoprotein; TG, triglyceride.

## 4 DISCUSSION

This study demonstrated that liver fat, assessed by CAP score, significantly reduced after a 3‐month structured lifestyle modification program in patients with MAFLD. In addition, weight reduction, which is the major determining factor for MALFD improvement and/or resolution, was achieved with the structured program and the CAP value may be used to monitor liver steatosis and respond to intervention. It is believed that this is the first study that evaluates the utility of CAP values for monitoring hepatic steatosis in an obese population with MAFLD through exercise and diet modification.

Excess calorie consumption, which leads to obesity and related comorbidities (e.g., metabolic syndrome), is the leading risk factor for MAFLD.^13^ Weight loss through structured programs aimed at lifestyle changes (e.g., a healthy diet and habitual physical activity) has been shown to effectively reduce obesity‐related liver steatosis and inflammation in patients with NASH in randomized controlled trials.^5^, ^14^, ^15^ In general, 7%–10% weight loss in overweight and obese patients with fatty liver disease results in improvement of liver enzymes and histology.^16^ In the present study, the average weight loss after 3 months of diet modification and increased physical activity was 7.74 kg, which corresponded to an overall weight loss of 8.8%. This resulted in a significant reduction in waist and hip circumference as well as body fat percentage. Concerning liver profile, AST (24.06 ± 8.85 U/L vs. 18.91 ± 6.4 U/L, *p* = .002) and ALT (33 ± 20.76 U/L vs. 23.72 ± 14.72 U/L, *p* = .012) was significantly improved after intervention, resulting in normalized mean levels. The current study reinforced knowledge of lifestyle modification associated with significant improvement in markers for liver enzymes and metabolic syndrome (e.g., lower blood pressure, cholesterol, and LDL). However, these changes were not evident in HbA1c, insulin, and fasting glucose levels or HOMA‐IR likely because none of the participants presented with T2DM.

The US Food and Drug Administration has recommended that therapeutic intervention for NAFLD should demonstrate evidence of histologic improvement of steatohepatitis without worsening fibrosis or improvement in fibrosis without worsening steatohepatitis, as evidenced by liver histology.^17^ However, liver biopsy is invasive and is associated with complications. In a recent meta‐analysis, percutaneous liver biopsy was associated with 2.4% and 9.5% of the major and minor complications, respectively, in patients with chronic liver disease.^18^ Therefore, noninvasive tests and imaging have been explored to monitor treatment response. TE (Fibroscan) has been widely used to measure hepatic steatosis in multiple chronic liver disease^19^, ^20^ The CAP cutoff score used in this study had an AUROC of 0.84, 0.86, and 0.93 for the diagnosis of S1, S2, and S3 disease, respectively.^11^ TE is available in most liver clinics and has become a point of care in the NAFLD or MAFLD population.^21^ Other imaging modalities include magnetic resonance imaging with proton density fat fraction measures the fat fraction in all segments of the liver and is accurate in detecting and quantifying the degree of steatosis in NAFLD.^22^ However, the technical complexity, limited availability, high cost, and contraindication for claustrophobic patients limit its practicality in the clinical setting.

Previously, CAP values were significantly decreased from baseline after 24 weeks of therapy in lobeglitazone‐treated NAFLD patients; however, the efficacy of lobeglitazone needs to be determined in further studies with the incorporation of liver histology.^21^ A recent study by Avcu et al. demonstrated that CAP is a reliable diagnostic tool for the early diagnosis of hepatic steatosis in obese patients with MAFLD.^23^ However, few studies show the utility of CAP in monitoring the response to intervention in MAFLD patients. Our study demonstrated that the CAP score has significantly reduced after 3 months of structured diet and physical activity program in patient with MAFLD, and majority of patients had reduction in steatosis severity at the end of the study.

The present study showed that the postintervention liver stiffness value did not decrease significantly in the MAFLD patients but trended toward statistical significance. This may be the result of a low‐fibrosis score (F0–F1) at baseline in most of the patients. In addition, this indicates that a longer study duration is needed to demonstrate changes in fibrosis score as the effect of weight loss on fibrosis appears to be smaller than that of the effect on steatosis. Future clinical trials should consider enrolling patients with a full spectrum of MAFLD severity with a longer study duration. The findings of the current study indicate the possibility of utilizing TE to monitor postintervention response in MAFLD patients.

The current study had several limitations. First, this is an uncontrolled experimental study that does not include a control group. This can be confounded by Hawthorne effect, which could lead to overestimate of the effectiveness of the intervention. Second, the study sample size was relatively small and recruited from a single tertiary medical center, which may be clinically different compared with that of the community or the general population. Furthermore, no histological data was noted regarding steatosis grade. Also, diagnostic variability may exist in TE, which is highly dependent on the technical skill of the examiners. However, histologic confirmation via liver biopsy is highly invasive and not recommended for this study. Finally, all participants were Chinese patients from Taiwan, and studies with patients from other ethnic groups are warranted.

In conclusion, this study suggests that short‐term lifestyle modification can effectively improve hepatic steatosis, and TE can be used to monitor therapeutic intervention in MAFLD population and may be introduced into clinical practice.

## CONFLICT OF INTEREST

All authors declare no conflict of interest.

## Supporting information


**Table S1** Distribution of patients with hepatic steatosis grade improvement after intervention.
