# Supplementary material for: Modifications decrease hepatic steatosis in Taiwanese with metabolic‐associated fatty liver disease
Source: Kaohsiung J Med Sci. 2022 Aug 22;38(10):1012–9. doi: 10.1002/kjm2.12580 (PMC11896208; doi:10.1002/kjm2.12580)
Supplement: Supplementary file 1 — Table S1 Distribution of patients with hepatic steatosis grade improvement after intervention. [file KJM2-38-1012-s001.docx]

**Supplementary Table**

**Table S1.** Distribution of patients with hepatic steatosis grade improvement after intervention.

|  |  | **After** | | | |  |
| --- | --- | --- | --- | --- | --- | --- |
|  |  | **S0** | **S1** | **S2** | **S3** | **Total** |
| **Before** | **S0** | 0 | 0 | 0 | 0 | 0 |
|  | **S1** | **5** | 3 | 1 | 0 | 9 |
|  | **S2** | **3** | **2** | 1 | 0 | 6 |
|  | **S3** | **2** | **2** | **7** | 6 | 17 |
|  | **Total** | 10 | 7 | 9 | 6 | 32 |

*p* = 0.001, S, steatosis grade; S0: <215, S1: 215-252, S2: 253-296, S3: >296.
